# Approach to the Patient With Bone Fracture: Making the First Fracture the Last

**DOI:** 10.1210/clinem/dgad345

**Published:** 2023-06-08

**Authors:** Mawson Wang, Markus J Seibel

**Affiliations:** The University of Sydney, Bone Research Program, ANZAC Research Institute, Concord, NSW 2139, Australia; The University of Sydney, Bone Research Program, ANZAC Research Institute, Concord, NSW 2139, Australia

**Keywords:** osteoporosis, secondary fracture prevention, primary care, fracture liaison service

## Abstract

The global burden of osteoporosis and osteoporotic fractures will increase significantly as we enter a rapidly aging population. Osteoporotic fractures lead to increased morbidity, mortality, and risk of subsequent fractures if left untreated. However, studies have shown that the majority of patients who suffer an osteoporotic fracture are not investigated or treated for osteoporosis, leading to an inexcusable “osteoporosis care gap.” Systematic and coordinated models of care in secondary fracture prevention known as fracture liaison services (FLS) have been established to streamline and improve the care of patients with osteoporotic fractures, and employ core principles of identification, investigation, and initiation of treatment. Our approach to the multifaceted care of secondary fracture prevention at a hospital-based FLS is illustrated through several case vignettes.

Osteoporosis is characterized by loss of bone mass and deterioration in skeletal microarchitecture, leading to weakness of the skeleton and increased susceptibility of fragility, or osteoporotic fractures. An estimated 1 in 3 women and 1 in 5 men older than 50 years will experience an osteoporotic fracture in their remaining lifetime (1). The personal and economic burden of fragility fractures is considerable, with subsequent disability, pain, loss of independence, and increased mortality in affected patients. Currently, an estimated 200 million people worldwide suffer from osteoporosis; with a rapidly aging population, the prevalence of osteoporosis and fragility fractures will continue to increase, profoundly affecting national health care systems.

Any osteoporotic fracture significantly increases the risk of subsequent fractures. Numerous studies have shown that the risk of subsequent fracture is highest in the first 1 to 2 years following an initial fracture, and then gradually decline over time (2-5). Mortality has also shown to be significantly higher than in the general population after most fractures, with the exception of radius fractures (6). Hip fractures are particularly associated with high mortality, with 1-year mortality rates ranging between 23% and 33% (7-10).

Unfortunately osteoporosis, even when diagnosed, often does not draw the same level of clinical urgency as other health conditions despite the known deleterious consequences on mortality and morbidity. Less than 20% of patients who experience an osteoporotic fracture are initiated on appropriate pharmacotherapy post fracture, in studies from the United States (11-13), Canada (14), Denmark (15), and Australia (16). In an Australian primary care setting, the rate of osteoporosis treatment in those with radiologically confirmed vertebral fractures was even lower, at 3.8%, even though vertebral fractures confer the highest risk of refracture (16, 17). Undiagnosed and untreated, up to 75% of such patients will sustain secondary fractures (18).

The undertreatment of osteoporosis persists despite widely available pharmacotherapy proven to be cost-effective and reduce the risk of subsequent fractures (19-21). The decision to investigate or treat patients for osteoporosis remains inconsistent and nonsystematic, with a previous study noting that lack of knowledge about for whom and when to intervene remains a substantial barrier to effective osteoporosis management (22). Low rates of appropriate treatment initiation following an osteoporotic fracture therefore constitute a considerable “osteoporosis care gap.”

Since the late 1990s, systematic and coordinated models of care in following up patients with osteoporotic fractures have emerged around the world (23, 24). These services, now known as secondary fracture prevention programs or fracture liaison services (FLSs), have been established in numerous hospital settings and may comprise multidisciplinary services combining hospital-based teams (eg, emergency, orthopedics, and orthogeriatrics), primary care, osteoporosis physicians (endocrinology and rheumatology), nurse coordinators, and allied health (physical therapist, occupational therapist, and dietitian). Most FLSs follow the original “3I” model of 1) identification, 2) investigation, and 3) initiation of appropriate treatment (25). Expansions to this model have been proposed by various researchers, including the “5I” model with additional domains in 4) improvement of adherence and 5) intelligence (26), and the “5IQ” model including 4) information, 5) integration with primary care, and 6) quality (27). FLS models that deliver the most intensive management approach are associated with reduced refracture risk (hazard ratio [HR] 0.18-0.67 over 2-4 years), reduced mortality (HR 0.65 over 2 years), increased bone mineral density (BMD) assessment (relative risk [RR] 2-3), and increased treatment initiation (RR 1.5-4.25) (28). A recent systematic review and meta-analysis confirmed that FLS care was associated with lower refracture risk (odds ratio 0.70) but only in studies with follow-up periods beyond 2 years (29). Another meta-analysis of FLSs has shown that programs with a more proactive and intensive approach to secondary fracture prevention are more effective in improving treatment initiation rates than interventions primarily based on patient or doctor education and are cost-effective (30). Globally, there are concerted efforts by various organizations in developing clinical standards for postfracture care, advocating for wider implementation of FLSs and benchmarking FLS performance outcomes (31).

Studies of FLSs in different global settings have shown it to be cost-effective or even cost-saving in comparison to usual care. A systematic review of 23 studies across North American, European, East Asian, and Australian FLS demonstrated Incremental Cost-Effectiveness Ratios ranging from $3023 to $28 800 US dollars per quality-adjusted life year (gained in Japan, to $14 513 to $112 877 US dollars in the United States (32). The higher figures estimated in American models were still below the threshold of $150 000 US dollars per quality-adjusted life year recommended in the United States (33). However, there have been no studies comparing the cost-effectiveness between hospital-based FLSs and general practitioner (GP)-based management.

The FLS at our tertiary hospital was established in 2004 and currently manages between 1000 and 1500 patients annually. Next we have included several clinical case vignettes based on real patients referred to our FLS, highlighting key aspects of a modified “5I” FLS model (Fig. 1).

**Figure 1.**
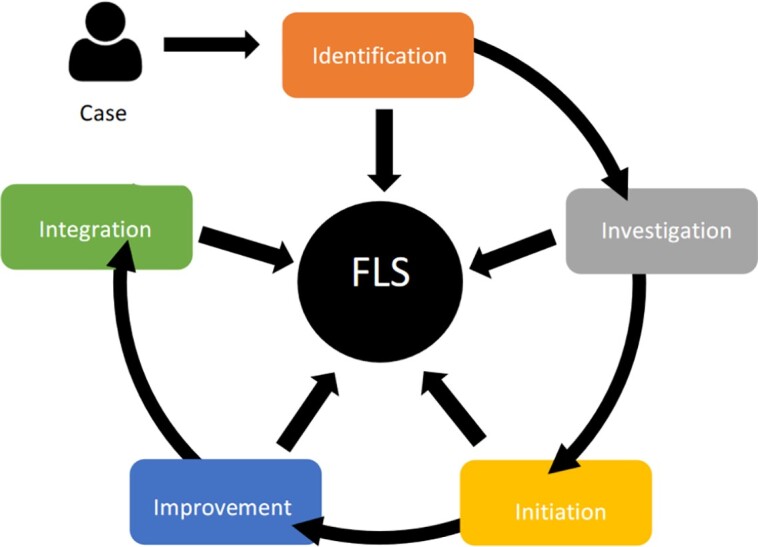
The five “I” model of secondary fracture prevention services. FLS, fracture liaison service.

##  

### Case 1—Identification

The use of an automated electronic search tool identifies an incidental T12 vertebral compression fracture in the report of a computed tomography scan undertaken for an unrelated indication in a 50 year-old man. The fracture is verified by the FLS coordinator and the patient is subsequently referred to the clinic for further assessment.

The patient's past medical history is extensive, including multiple risk factors for osteoporosis. He is a current smoker of 30 pack-years and previously consumed excessive alcohol for several decades but is now abstinent following a diagnosis of liver cirrhosis. He has been on repeated short courses of prednisolone for exacerbations of chronic obstructive pulmonary disease. On further questioning, he reports chronic steatorrhea, raising the possibility of pancreatic exocrine insufficiency secondary to alcohol excess. He consumes around one dietary serving of calcium daily and is not on calcium or cholecalciferol supplementation. There is no unintentional weight loss. He is employed as a yardhand and does not engage in other forms of regular physical activity. There is intermittent lower thoracic back pain corresponding to his vertebral fracture, exacerbated by forward flexion. Physical examination of his lower limbs is normal for power, sensation and reflexes, and he reports no paresthesiae.

A dual-energy x-ray absorptiometry (DXA) scan performed demonstrates L2 to L4 BMD 0.914 g/cm^2^, T score −2.7 SD, and left femoral neck BMD 0.776 g/cm^2^, T score −2.3 SD. His secondary osteoporosis screen reveals normal renal function with estimated glomerular filtration rate (eGFR) greater than 90 mL/min/1.73 m^2^ (≥60), corrected calcium 2.49 mmol/L (2.10-2.60 mmol/L), 25-hydroxyvitamin D 73 nmol/L (≥50), parathyroid hormone (PTH) 5 pmol/L (1.6-6.9 pmol/L), thyrotropin (TSH) 1.08 mIU/L (0.27-4.20 mIU/L), testosterone 21.1 nmol/L (10.0-30.0 nmol/L) with normal luteinizing hormone and follicle-stimulating hormone. Bone resorption and formation markers C-terminal telopeptide of type I collagen (CTX-I) and aminoterminal procollagen type I propeptide (PINP) are within normal limits. Celiac and myeloma screens are negative.

Using an electronic case-finding tool, we have captured a high-risk patient whose osteoporosis would likely have been missed otherwise. The patient has multiple medical and lifestyle risk factors for osteoporosis. He is encouraged to cease smoking, continue abstinence from alcohol, increase dietary calcium intake to 3 servings daily, maintain adequate sunlight exposure, and commence weight-bearing exercises. His GP was asked to investigate his possible pancreatic exocrine insufficiency. We recommend commencement of yearly intravenous zoledronic acid and he receives his first dose in the clinic. He will likely require several years of antiresorptive therapy.

To be efficient, any secondary fracture prevention program needs to be able to systematically identity patients with a recent osteoporotic fracture at or soon after their presentation and arrange for timely follow-up. Hospital-based referral pathways may be established whereby patients presenting with a fracture are referred to the FLS from the emergency department, orthopedic, orthogeriatric, or other medical teams with specified inclusion and exclusion criteria. A designated FLS coordinator assumes responsibility for the identification and triaging of such patients (23, 25). Their role may include patient education, communication between multidisciplinary teams, obtaining history and risk assessment, arranging DXA scans and other investigations, data collection, arranging FLS follow-up, and monitoring treatment adherence.

Nevertheless, this type of identification process has its limitations, being not only labor and resource intensive but also failing to capture a large proportion of cases (34). In recent years, the integration of “artificial intelligence” into the FLS model has assisted with the identification of patients with fractures, including and in particular machine-based natural language processing (NLP) tools capable of analyzing linguistic data from electronic health records, radiology reports, clinical coding, and discharge summaries. Grundmeier et al (35) used NLP methods to identify pediatric long-bone fractures within emergency department radiology reports, and these performed better than traditional diagnostic coding. Newer algorithms have included skeletal site-specific rules, further improving diagnostic accuracy (36). There are systems that implement NLP in parallel to voice recognition software during radiologist reporting (37). Within Sydney there have been 2 NLP tools created for the purpose of fracture detection; an automated electronic search tool that interrogates disease coding within electronic records and text-based searches of imaging reports (34), and an X-Ray Artificial Intelligence Tool (XRAIT), which focuses on patterns of words in radiology reports (38). XRAIT was shown to detect 3 times more fractures compared with manual case finding by a staff member, while maintaining high specificity (38). Comparison of the 2 electronic search programs revealed generally high positive predictive value for detecting minimal trauma fractures, identifying slightly different subsets of patients but still missing some osteoporotic fractures (34). There has also been substantial progress in the development of tools trained to detect fractures across multiple imaging modalities with high diagnostic accuracy (39, 40). Cases 1 and 5 illustrate the clinical utility of an electronic case-finding tool in identifying patients at high risk of secondary fracture who otherwise may have been overlooked with existing systems.

Future directions in electronic search tools may see a hybridization of visual recognition and language search capabilities with increasing accuracy and precision, and will likely substantially improve the detection of patients with osteoporotic fractures and offer an opportunity for optimal link-up to FLS.

### Case 2—Investigation

A 54-year-old perimenopausal woman is referred to the clinic by the emergency department following a right distal radius fracture sustained after slipping on a wet tiled surface. Her fracture is managed conservatively in a cast. She has a background of iron-deficiency anemia but is otherwise healthy and not on any prescription medications.

In terms of risk factors for osteoporosis, her mother has low BMD without major fractures. The patient is currently perimenopausal. She is an ex-smoker of around 2 pack-years and consumes 1 to 2 standard drinks per week. There are no other risk factors including corticosteroid use or thyroid or celiac disease. Physical activity is in the form of walking and Zumba classes several times per week. She takes cholecalciferol 1000 IU daily without a calcium supplement but consumes milk and cheese regularly.

Her DXA scan in clinic demonstrates L1 to L4 BMD 1.019 g/cm^2^, T score −0.3 SD, left femoral neck BMD 0.759 g/cm^2^, T score −0.8 SD and left total hip BMD 0.874 g/cm^2^, T score −0.6 SD. A thoracolumbar spine x-ray does not demonstrate evidence of vertebral compression fractures. Her secondary osteoporosis screen is normal.

This is a younger perimenopausal woman who presents with a distal radius fracture without evidence of osteoporosis on DXA scan. It is possible that her fracture was related to the mechanism of her fall. However, it is important to note that a third of hip fracture patients previously suffered a radial fracture (41). Thus, radial fractures should be considered a sentinel event and thoroughly investigated. In our present patient, appropriate workup has shown no evidence of occult vertebral compression fractures on thoracolumbar spine screening, and her secondary osteoporosis screen is negative. Her absolute fracture risk is currently low because of her young age, normal BMD, and lack of osteoporosis risk factors. She is counseled on lifestyle and dietary modifications rather than commenced on pharmacotherapy.

The purpose of further FLS-initiated investigations is to assess and estimate future fracture risk. Risk factors for fractures, including advanced age, female sex and postmenopausal status, prevalent fractures, falls, parental history of hip fracture, smoking, corticosteroid use, alcohol excess, and numerous other secondary causes of osteoporosis should be explored (Table 1), particularly in patients with DXA Z scores less than −2 SD and multiple previous fractures (42). These risk factors are incorporated in validated web-based absolute fracture risk calculators such as the Fracture Risk Assessment Tool (FRAX) (43) and the Garvan Fracture Risk Calculator (44, 45). A targeted physical examination should examine for evidence of vertebral fractures (eg, thoracic kyphosis, focal vertebral tenderness, increased wall-to-occiput distance > 0 cm, height loss) and causes of secondary osteoporosis (eg, hyperthyroidism, Cushing syndrome, hypogonadism).

**Table 1.** Secondary causes of osteoporosis

Medications	Glucocorticoids Aromatase inhibitors Depot medroxyprogesterone acetate Antiepileptics Antiretroviral therapy Thiazolidinediones
Endocrine disorders	Hyperthyroidism Hyperparathyroidism Hypogonadism (estrogen or testosterone deficiency) Growth hormone deficiency Acromegaly Diabetes mellitus Vitamin D deficiency Hypercalciuria
Rheumatological/inflammatory disorders	Rheumatoid arthritis Systemic lupus erythematosus Ankylosing spondylitis Connective tissue disorders
Hematological/oncological disorders	Multiple myeloma/monoclonal gammopathy of undetermined significance Thalassemia major Systemic mastocytosis
Gastrointestinal disorders	Celiac disease Inflammatory bowel disease Postbariatric surgery, gastrectomy Malnutrition/caloric restriction Liver cirrhosis
Other disorders	Transplant recipients Prolonged immobilization
Lifestyle factors	Smoking Alcohol Pregnancy and lactation

DXA scans should be used for initial assessment of BMD and subsequent monitoring. These may not be feasible in all patients because of technical issues (joint replacements or severe degenerative changes in the measurement area, extremes of body mass index, inability to maintain correct positioning for scan), nor is it necessarily required in patients for whom it would unlikely alter risk assessment or decision for treatment. Quantitative computed tomography may offer a more accurate assessment of BMD than DXA in patients with severe degenerative changes, abdominal aortic calcification, and extremes of body mass index; however, serial measurements are not recommended because of the relatively high radiation exposure (46).

It is practice for our FLS to perform serum creatinine (intravenous bisphosphonates are contraindicated at eGFR < 35 mL/min/1.73 m^2^), calcium and phosphate, parathyroid hormone, 25-hydroxyvitamin D, TSH, celiac serology, serum electrophoresis, immunofixation, and free light chains, although other recommendations may be less comprehensive. In men, total testosterone should be considered if the patient's history is suggestive of hypogonadism (47), and female reproductive hormones in anovulatory premenopausal females (48). Further targeted investigations may be undertaken depending on clinical suspicion of secondary causes of osteoporosis. Finally, as more than half of patients with a vertebral fracture may be asymptomatic, thoracolumbar spine x-ray or vertebral fracture assessment should be offered as part of osteoporosis screening (42).

### Case 3—Initiation

An 86-year-old woman trips while gardening at home, landing on her right hip. She presents to the emergency department, is diagnosed with a right neck of femur fracture, and managed with a right total hip replacement. She has a background of osteoarthritis resulting in a previous left hip replacement, hypertension, and hypercholesterolemia. Postoperatively she is jointly managed by the orthopedic and orthogeriatrics teams. The “Concord Post-Hip Fracture Osteoporosis Treatment Protocol” (49) is initiated by the orthogeriatrics team: renal function, serum calcium, and 25-hydroxyvitamin D levels are measured and as the patient's 25-hydroxyvitamin D level is less than 50 nmol/L, she is given 50 000 IU of cholecalciferol daily for 5 days. This corrects her vitamin D deficiency and she receives a single dose of intravenous zoledronic acid 4 mg on day 6 post surgery. She is discharged on a cholecalciferol supplement along with a referral to be seen by the Concord FLS in 12 months’ time.

One year later, she is reviewed at the FLS for follow-up as part of the protocol. She now mobilizes with a walker at home and has not had further falls, but is largely housebound. She consumes almost no dietary calcium and has stopped taking her cholecalciferol supplement prescribed at time of discharge. She reports no family history of osteoporosis, and underwent menopause in her 50s. She is a lifelong nonsmoker, does not consume alcohol, and has no other osteoporosis risk factors.

A DXA scan is not arranged because of the presence of bilateral hip replacements and severe degenerative spine disease. Pathology results indicate normal renal function with eGFR 80 mL/min/1.73 m^2^, corrected calcium 2.33 mmol/L, PTH 8 pmol/L, and 25-hydroxyvitamin D 67 nmol/L. Serum protein electrophoresis, serum free light chain, and celiac serology are all within normal limits. Serum CTX-I is in the low normal range in the context of receiving zoledronic acid 1 year ago.

This older patient has sustained a minimal trauma hip fracture and is at high risk of future osteoporotic fractures. She is switched from intravenous zoledronic acid to subcutaneous denosumab in light of her poor functional status and advanced age, and instructed to continue this lifelong without dose interruption. She is recommenced on calcium with cholecalciferol supplement daily. After a hip fracture, the risk of subsequent fractures compared to those without hip fractures increases to 2.5 times in women and almost 5 times in men (2). Rather than provide a recommendation to the patient's GP to consider osteoporosis therapy following hospital discharge, our Post-Hip Fracture Osteoporosis Treatment Protocol adopts a proactive approach in early initiation of osteoporosis treatment during their index admission and provides link-up with FLS for ongoing treatment and review.

As previously described, the majority of patients are not diagnosed or treated for osteoporosis following a minimal trauma fracture, including hip fractures. A study from a US veterans’ hospital demonstrated that even among the cohort with minimal comorbid conditions, only 13% of the study population received pharmacotherapy following a hip fracture (13). The Australian & New Zealand Hip Fracture Registry (ANZHFR) estimated that only 38% to 45% of hip fracture patients were on appropriate pharmacotherapy at 120 days post discharge (50).

In 2019, the ANZHFR report identified our tertiary hospital as having some of the lowest rates of predischarge osteoprotective therapy in patients admitted for a hip fracture. In response to this alarming data, the “Concord Post-Hip Fracture Osteoporosis Treatment Protocol” was developed as a collaboration between Geriatric Medicine, Endocrinology, Orthopaedic Surgery, Pharmacy, and Nursing. The protocol targets patients with minimal trauma hip fractures not currently on osteoporosis therapy. A small set of laboratory tests is ordered to ensure the patient has sufficient renal function (eGFR > 35 mL/min/1.73 m^2^), normal serum calcium, phosphate, and 25-hydroxyvitamin D levels prior to administering intravenous zoledronic acid before discharge (49). The 20% of patients who are vitamin D deficient receive 50 000 IU of cholecalciferol for 5 consecutive days to normalize their serum 25-hydroxyvitamin D levels before receiving the bisphosphonate. This type of targeted inpatient initiation of treatment ensures that pharmacotherapy is not delayed due to awaiting DXA scans or outpatient appointments. Another integral component of the protocol is making a 12-month appointment with the FLS before discharge, thus reducing the chance of patients being lost to follow-up.

Initiation of appropriate osteoprotective treatment is thus a key component of secondary fracture prevention. The most intensive or “Type A” FLS model, with a focus on identification, investigation, and initiation of osteoporosis treatment, leads to around 46% of patients being initiated on treatment, compared to only 8% in “Type D” models, in which only patient education is provided without further action (30). In a randomized controlled study, Ganda et al (51) demonstrated that following initiation of oral antiresorptive therapy through the FLS, 2-year treatment compliance was similar regardless of whether patients continued follow-up in the program or were discharged back to their GP. This highlights the importance of overcoming the “therapeutic inertia,” as once treatment has been initiated, ongoing GP care is as effective as FLS in maintaining treatment compliance. Appropriate initiation of pharmacotherapy has been proven to reduce future fracture risk. There is relative risk reduction of up to 39% to 44% for vertebral fractures and 20% to 36% for nonvertebral fractures with oral bisphosphonates; 46% to 70% for vertebral fractures and 25% to 27% for nonvertebral fractures with zoledronic acid; 68% for vertebral fractures and 20% for nonvertebral fractures with denosumab; 65% for vertebral fractures and 53% for nonvertebral fractures with teriparatide; and 73% for vertebral fractures and 36% for nonvertebral fractures with romosozumab (42).

### Case 4—Improvement

An 80-year-old woman is referred to the FLS clinic after imaging performed for acute back pain revealed atraumatic T11 to L1 vertebral compression fractures. Five years ago, she was commenced on subcutaneous denosumab 60 mg every 6 months by another practitioner on the basis of low BMD in the absence of minimal trauma fractures. She has adhered to this therapy for 3 years but reads about the rare side effect of atypical femoral fractures and decides to cease treatment. Unfortunately, she is recently diagnosed with T11 to L1 vertebral compression fractures possibly as a result of the rebound phenomenon seen in cessation of denosumab (52-55).

Her other comorbidities include type 2 diabetes and hypertension. She is reasonably active with walking and is adherent to a combined calcium and cholecalciferol supplement. There are no active dental issues. She has a slightly kyphotic posture with resolving tenderness at the site of her vertebral fractures.

A DXA scan in clinic shows L2 to L4 BMD 0.612 g/cm^2^, T score −4.2 SD, left femoral neck BMD 0.475 g/cm^2^, T score −3.4 SD, and left total hip BMD 0.641 g/cm^2^, T score −2.5 SD, representing a greater than 6% decline over the past 3 years. Pathology results show stable chronic renal impairment with eGFR of 64 mL/min/1.73 m^2^, normal calcium, PTH, 25-hydroxyvitamin D, TSH, and a negative myeloma screen. The bone resorption marker CTX-I is at the upper end of the reference range.

This patient has sustained several vertebral compression fractures following cessation of denosumab. We explain to her and provide a clear letter communicating to her GP that denosumab cannot be ceased abruptly because of the risk of rebound increased bone turnover and the ensuing decline in BMD and development of multiple vertebral fractures. We explore the patient's concerns regarding long-term side effects of antiresorptive therapy, and offer alternative therapy such as annual zoledronic acid infusion to improve adherence. The patient prefers to continue denosumab injections and we administer her next dose in the clinic, and provide a prescription to continue her next dose in 6 months with her GP. She will return for follow-up in 1 year to ensure ongoing treatment adherence, and no further fractures have occurred.

A key role of the FLS is to ensure and improve adherence to osteoprotective pharmacotherapy and provide clear patient information. Ganda et al (56) reported on predictors of refracture among patients managed within an FLS and found that a low medication possession ratio of 50% or less (a surrogate marker for adherence) was a significant risk factor. Barriers to continuation of therapy should be explored and managed, such as cost, convenience of administration, and side effects, and perceptions toward osteoporosis. If adherence to a weekly oral bisphosphonate is difficult, consider switching to a monthly bisphosphonate, annual intravenous bisphosphonate, or subcutaneous denosumab every 6 months. Results from meta-analyses suggest that effective education within an FLS increased medication adherence by 22% compared to controls (57). Noninitiation of treatment is a strong predictor for poor adherence to clinical follow-up (58). As this case has illustrated, patient concerns surrounding current or long-term side effects of pharmacotherapy should be identified and managed to avoid inadvertent cessation of therapy.

Atypical femur fractures (AFFs) are atraumatic fractures of the subtrochanteric femoral shaft, believed to result from accumulation of microdamage caused by the inhibition of bone remodeling during prolonged antiresorptive therapy (59). While the incidence of AFFs rises with increasing duration of treatment, with an absolute risk of around 11 fractures per 10 000 person-years of bisphosphonate treatment and similar risk in denosumab users, the absolute risk remains very low and the benefit of treatment usually outweighs the risk of AFF by a large margin (60-62). As the risk of AFF declines rapidly following cessation of antiresorptive therapy, a “drug holiday” may be considered in appropriate patients, that is, in those without major risk factors such as prevalent or new fragility fractures or very low BMD (61, 63). In stark contrast, denosumab discontinuation is not recommended at any point as this leads to a complete and rapid reversal of its effects on bone turnover and BMD, with an increased risk of multiple vertebral fractures (53-55, 64). Risk factors for multiple vertebral fractures post denosumab cessation include longer duration of treatment and cessation, prevalent vertebral fractures, greater hip BMD gain on treatment, and greater hip BMD loss off treatment (52, 53). Thus, while adherence to all forms of osteoprotective pharmacotherapy is important, particular attention should be paid to 1) communicate clearly to patients and their GPs that once denosumab is commenced the drug cannot be ceased without subsequent antiresorptive therapy to prevent bone loss, and 2) carefully consider the appropriateness of using denosumab in a younger patient in whom lifelong antiresorptive treatment is neither desirable nor indicated.

Osteonecrosis of the jaw, another extremely rare adverse event associated with antiresorptive therapy, typically occurs in the setting of invasive dental procedures and higher-dose regimens in oncology patients (65). Patients should be aware that the incidence of osteonecrosis of the jaw in the setting of osteoporosis treatment is only around 1 in 10 000 to 100 000 patient-years (66). They should be counseled on the importance of informing their dentist of antiresorptive treatment, with antiresorptive therapy recommended 4 to 6 weeks after invasive procedures to promote wound healing (67).

### Case 5—Integration With Primary Care

A 75-year-old man is referred by his GP to a private radiology practice for a chest x-ray to investigate a cough. An electronic search tool, employed by a staff member at the radiology practice, scans all radiology reports and detects mention of several rib fractures in the radiology report. The GP receives the chest x-ray report via the usual communication methods with the radiology practice. Several days later, the GP also receives a separate message within their clinical software, alerting them of their patient's recent rib fracture and to encourage further investigations into whether this is an osteoporotic fracture requiring treatment. Accessible links to osteoporosis management guidelines are provided.

When the patient attends his GP for postdischarge follow-up, the GP discusses the need for further investigations for osteoporosis and refers the patient for a DXA scan. The result indicates reduced bone density with lowest T score −2.6 SD at the left femoral neck. A secondary osteoporosis screen is ordered. Several risk factors for osteoporosis are uncovered during history-taking, including corticosteroid treatment for polymyalgia rheumatica, insufficient calcium intake, and 25-dihydroxyvitamin D deficiency. The patient is also noted to have frequent falls and is referred to the local Strength Training, Rehabilitation and Outreach Needs in Geriatric Medicine service for a supervised, tailored outpatient exercise program. The patient is commenced on subcutaneous denosumab injections every 6 months and a combined calcium and cholecalciferol supplement.

One of the limitations of an FLS is its low capacity to manage all patients diagnosed with osteoporotic fractures. There is growing consensus that the majority of osteoporosis cases should be managed within primary care rather than in underresourced hospital-based specialist services (68). Most hospital-based FLSs would be overwhelmed if they were to identify and manage a several-fold increase in patients as a result of using improved case-detection tools. There is currently little meaningful engagement and integration between FLS and primary care, with inadequate “safety net” mechanisms to identify patients who become lost between the fracture event and commencement of osteoporosis care. Interventions aimed at targeting and involving primary care in the management of secondary fracture prevention have been shown to be effective. A systematic review and meta-analysis of interventions improving osteoporosis management in primary care found that most approaches were multifaceted and involved a combination of sending GP notifications, providing patients with educational material, providing GPs with osteoporosis training, and phoning patients to ensure initiation of further management (69). Such interventions increased the incidence of BMD testing and/or pharmacotherapy initiation in high-risk patients by up to several times (70-73). A review of several Spanish FLSs identified strategies to further improve integration with GPs, including use of shared software between FLSs and GPs to facilitate communication of patient reports, and FLS-led training sessions at GP centers (74). Nevertheless, such interventions are labor intensive and currently still the exception rather than the norm.

Future directions in improvement of existing FLS models should consider effective integration with primary care providers, particularly at the initial stage when a fracture is first identified. Our team is working on the implementation of a model, illustrated earlier, which aims to establish primary care as the center of osteoporosis management, with integration from hospital-based FLSs and community-based radiology practices serving as mechanisms of case identification and referral to the GP. Models of FLS care that integrate primary care into secondary fracture prevention are urgently required to help with closing the osteoporosis care gap.

## Conclusion

Any fragility fracture should be considered as a sentinel event that requires close evaluation, investigation, and decision-making regarding the need for further treatment. FLSs have not only been shown to be effective in reducing the risk of refracture compared with standard care, but are also cost-effective. The modified “5I” model we have presented—“identification, investigation, initiation, improvement and integration”—is only one of various models an FLS may adopt. Hospital-based FLSs alone cannot manage the growing burden of osteoporosis, and improved integration with primary care will be essential in the years to come.

## Data Availability

No data sets were generated for this paper.

## Abbreviations

AFF: atypical femur fracture
ANZHFR: Australian & New Zealand Hip Fracture Registry
BMD: bone mineral density
CTX-I: C-terminal telopeptide of type I collagen
DXA: dual-energy x-ray absorptiometry
eGFR: estimated glomerular filtration rate
FLS: fracture liaison service
GP: general practitioner
NLP: natural language processing
PTH: parathyroid hormone
TSH: thyrotropin
XRAIT: X-Ray Artificial Intelligence Tool
